# Use of corn stover as bulking agent in dairy manure composting toward Japanese circular dairy farming

**DOI:** 10.1371/journal.pone.0241064

**Published:** 2020-10-22

**Authors:** Koki Maeda

**Affiliations:** NARO, Hokkaido Agricultural Research Center, Dairy Research Division, Sapporo, Japan; Kyonggi University, REPUBLIC OF KOREA

## Abstract

In response to the recent development of ear corn feeding systems and the shortage of bulking agents for manure composting in the Hokkaido region, the plausibility of using corn stover (a residue of ear corn harvesting) as an alternative bulking agent for dairy manure composting was tested. The temperature profile, quality of the final product, and greenhouse gas emissions were evaluated and compared with the values obtained from manure that used wheat straw, a typical bulking agent. A sufficiently high temperature profile (>70°C) and active organic matter degradation were achieved by mixing in corn stover. After active organic matter degradation for 8 weeks, CO_2_ production was significantly lower and the stable final products were obtained. The total solids level increased significantly, to 48.8–50.4%, while the C/N ratio dropped significantly, from 19.9–21.8 to 11.2–12.8. Methane emission from the corn-stover-based pile was 0.36% of initial volatile solids, while nitrous oxide emission was 0.58% of initial N, proving that the use of corn stover can mitigate greenhouse gas emission and that its mitigating effect was comparable to those of other bulking agents. Together, the results showed that the use of corn stover can be a suitable alternative bulking agent for dairy manure composting and can serve as part of an ecologically friendly and “circular” method of dairy farming.

## Introduction

Corn is one of the major feed crops for the dairy industry. Its current cultivation area in Japan is 94,600 ha, of which 59% is in Hokkaido. Modern Japanese dairy farmers depend heavily on imported feed: 76% of roughage and 88% of concentrated feed are imported (total digestible nutrient base; [1]). Due to increases in prices of internationally traded feed grain, the import value has doubled within 20 years, from 33,173 yen t^-1^ (2000) to 66,604 yen t^-1^ (2019), posing a severe management problem for Japanese livestock farmers [1]. Therefore, it is a challenge for the Japanese dairy industry to increase their self-sufficiency in concentrate feed for sustainable milk production. From this point of view, production of ear corn silage may be a solution because of its high yield, nutrient content and palatability. Therefore, some experimental trials of ear corn silage production are under way in the Japanese dairy industry [2]. As a result, corn stover as a by-product of ear corn production is expected to increase in this region.

Because corn harvesting leaves stover in the field, the adequate use of stover is very important for local farming systems. For example, it can be mixed into the soil to maintain soil organic carbon or to improve soil physical properties and crop production in subsequent years [3–5]. Alternatively, stover can be collected to generate renewable energy, such as biogas production through anaerobic digestion [6,7], conversion to bioethanol [8], or both [9].

An alternative use of corn stover is as a bulking agent for manure composting. Composting is the main manure management system for the Japanese dairy industry, and the use of a bulking agent is crucial in composting. It provides a complex physical structure that provides free air space and air flow into the pile inside [10–12], enabling microbes to uptake oxygen for efficient organic matter degradation. Various agricultural residues, such as rice/wheat straws, sawdust, and chaff, are used as bulking agents in manure composting [13–16]. However, because the availability of bulking agents in Hokkaido is limited, corn stover can solve two problems: low self-sufficiency of concentrate feed by enhancing ear corn production and running out of bulking agent in manure composting.

In this context, the objective of this study was to assess the validity of corn stover as an alternative bulking agent for dairy manure composting in the Hokkaido region. To do this, here we evaluated both the collection of ear corn stover by applying a commercial roll baler system and the use of stover for dairy manure composting. Because it is known that manure compost can be a source of greenhouse gases (GHGs) such as methane (CH_4_) and nitrous oxide (N_2_O) [17], the effects of corn stover as bulk on GHG emissions during composting were also investigated in order to assess the environmental impact.

## Materials and methods

### Stover pickup and calculation of operating efficiency

Maize variety P9400 (Pioneer Hi-Bred Japan Co., Ltd.) was planted in an experimental field (1.4 ha) at Hokkaido Agricultural Research Center (HARC; Sapporo City, Hokkaido) at a planting density of 91,860 ha^-1^. After the ear corn was harvested, the stover was left in the field for 2 weeks and then collected using a roll baler with variable diameter (working width 1.9 m, nominal diameter 0.9~1.8/ 0.9~1.55×1.2 m). Two experimental plots, one with and one without the use of a gyro rake (working width 5.4 m) before collection, were used to test whether the rake could increase collection efficiency. The operating efficiency was estimated based on the time used and the amount of stover collected.

Stover was collected from the field manually within five randomly set squares (9.2 m^2^). Moisture content was measured after the stover was dried at 60°C for 48 hours to estimate the amount of stover (dry weight) left in the field. The amount of stover collected by the roll baler was estimated by the fresh weights, diameters, and widths of the rolls. The collection efficiency was calculated using these parameters.

### Composting experiment

The composting experiment was performed twice at HARC. Ear corn stover was dried under ambient air before use. The cows were fed orchard grass silage and corn silage, oat hay, alfalfa hay, beet pulp, and two types of concentrate mixtures to meet their digestible energy requirements, as recommended by the Japanese Feeding Standard for Dairy Cattle. All animals received humane care as outlined in the Guide for the Care and Use of Experimental Animals (Animal Care Committee, National Agricultural Research Center for Hokkaido Region) as well as the Guidelines for Proper Conduct of Animal Experiments (Science Council of Japan, June 1, 2006). The compost in this study consisted of excrement from lactating Holstein cows and corn stover or wheat straw.

About 4 t of dairy cow excrement and 150 kg of each of the two types of bulking agents (corn stover, CS; wheat straw, WS) were mixed and then piled up on a waterproof concrete floor. Each mixture was piled up into a conical shape approximately 1.6 m in height and 3 m in radius at the beginning of the experiment. The mixtures were weighed on a truck scale. The piles were turned once every 2 weeks by a front loader and manure spreader in order to introduce fresh air into the pile for better organic matter degradation. The temperatures in the compost piles and of the ambient air were measured hourly using a Thermo Recorder RTW-30S (Espec, Japan). Fresh compost samples were taken at the start and end of the three composting experiments and just after each turning with complete homogenization.

Gaseous N_2_O, CH_4_, NH_3_, and CO_2_ emissions were measured using a dynamic chamber system and an infrared photoacoustic detector (IPD; INNOVA, Denmark) as described previously [18]. The chamber system consisted of a polyvinyl chloride (PVC) chamber equipped with an air-blowing ventilator and a gas sampling port on the ventilation exhaust. The chamber was 4 m in width, 6 m in depth, and 4 m in height. Four vent holes 10 cm in diameter were installed in the upper part of the chamber, and each was connected to a PVC pipe running to the ventilation blower installed outside the chamber. An inverter kept the airflow constant throughout the experimental period. Fresh air was introduced under the skirt of the chamber. The air was sub-sampled using a Teflon tube (4 mm in diameter) inserted just before the in-line fan. The gas concentrations of exhaust air were measured every 30 min in two replications. The respective rate of emission (E) was estimated according to Fukumoto et al. [19]. The gas concentration of the inlet air was subtracted from that of the outlet air and then multiplied by the ventilation rate.

### Chemical analysis of the compost

Fresh compost samples, each weighing about 500 g, were collected at the start and end of the composting experiments and just after each turning. The samples were homogenized, and fresh subsamples were used to measure total solids, volatile solids, inorganic-N, pH, and electrical conductivity, or were stored at -20°C for total nitrogen determination. Total solids (TS) were measured after the samples were dried overnight at 105°C, and the dried samples were powdered and used for C/N ratio determination. Volatile solids (VS) were measured after the samples were processed at 600°C for 1 h. The C/N ratio was determined using a C/N analyzer (vario MAX CNS; Elementar, Germany).

To measure inorganic-N, pH, and electrical conductivity, 5 g of fresh compost was placed into a 50 ml polypropylene tube with 40 ml of deionized water, then shaken (200 rpm, 30 minutes) and centrifuged (3,000 g, 20 minutes). The supernatant was collected and NH_4_^+^, NO_2_^—^N, and NO_3_^—^N were measured using ion chromatography (ICS-1600; Dionex, USA); pH and electrical conductivity (EC) were determined with calibrated electrodes (Horiba, Japan).

### Statistical analysis

The chemical components were analyzed by ANOVA using the general linear model procedure described by SAS [20]. Tukey’s multiple range comparison tests were used to separate the means. A value of P<0.05 was considered statistically significant.

## Results

### Stover collection efficiency

The amount of stover left in the field on harvest day was 9,568 kg ha^-1^, which dropped to 7,612 kg ha^-1^ after 2 weeks due to drying in the field. In line with this, the moisture content decreased significantly, from 76.0% to 59.2%, during this period. The amount of corn stover collected and the operational efficiency are summarized in Table 1. The average collection rate with the gyro rake was 2,016 kg ha^-1^, accounting for 26% of the stover left in the field. The recovery rate with only the roll baler was 1,643 kg ha^-1^, accounting for 22%. Collection with the gyro rake was more economically feasible, since the operation time was lower (0.87 h ha^-1^) compared to the roll baler only (1.66 h ha^-1^) and its diesel consumption was only half (19 L) that of the roll baler only (38 L). Although the use of the gyro rake decreased both operation time and diesel consumption, thus making it economically more feasible, it also increased the mud content in the rolls significantly.

**Table 1:** Summary of the corn stover pickup operation.

	Amount of picked-up stover (dry weight)		Pickup efficiency	Operation time	Operation manpower	Diesel used
	(kg ha^-1^)	(m^3^ ha^-1^)	(%)	(h ha^-1^)	(person)	(L)
With gyro rake	2,016^a)^	14.5	26	0.87	2	19
Without gyro rake	1,643^b)^	12.9	22	1.66	1	38

a) n = 2

b) n = 1.

### Composting experiments

**Fig 1:**
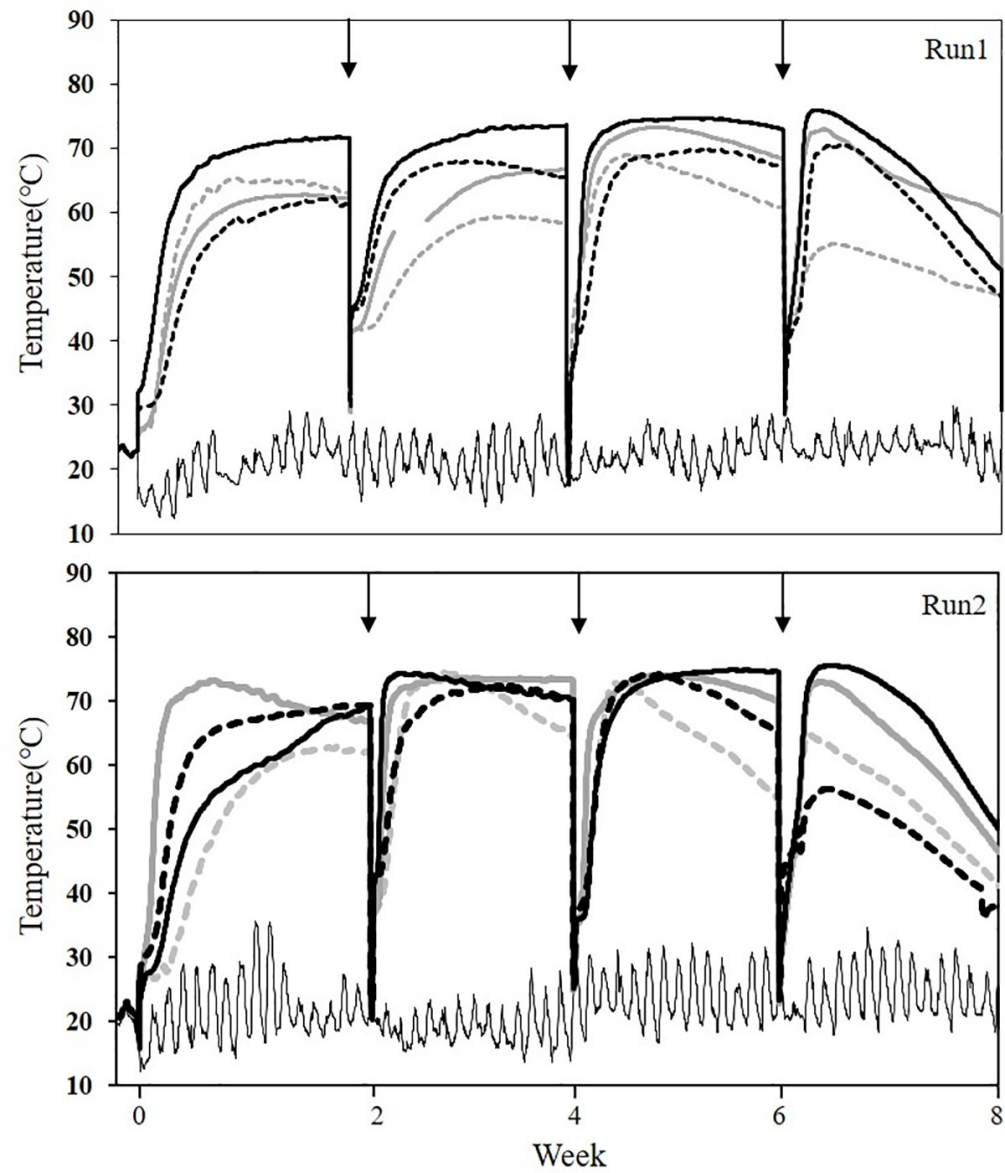
Temperature profiles of compost piles. Black solid lines, CS core; black dashed lines, CS bottom; light grey bold lines, WS core; light grey dashed lines, WS bottom; thin black lines, ambient temperature. Arrows indicate pile turnings.

Temperature significantly increased immediately after piling, reaching 75.7±0.2°C in CS piles and 73.7±0.5°C in WS piles (Fig 1). As a result of active organic matter degradation, the initial weight of the CS piles (4,105±7.0 kg) decreased to 985±21.2 kg. Of the initial mass in the CS piles, 76.0% was degraded, mostly as CO_2_ and water vapor. This was comparable to the WS piles, which showed a significant decrease, from 4,085±49.5 kg to 1,000±127.3 kg, after the 8-week composting process. The chemical and physical properties of the initial and final compost are shown in Table 2. TS increased significantly, from 24.8±4.2% to 49.6±1.1% in CS piles and from 24.8±1.7% to 40.7±7.7% in WS. Active organic matter degradation is also reflected in the C/N ratio, which decreased significantly, from 20.9±1.4 to 12.0±1.1 in the CS piles and from 19.9±0.2 to 10.6±0.5 in WS. These differences were not statistically significant, indicating that active organic matter degradation was very similar with either bulking agent and that either material can contribute to a successful active composting process.

**Table 2:** Chemical parameters of the compost.

Time	Bulking agent	Run	Weight	TS		VS		pH		EC		NH_4_^+^-N		NO_2_^-^-N		NO_3_^-^-N		TN	C/N ratio	
			kg	%	sd	%TS	sd		sd	mS cm^-1^	sd	mg kg^-1^ TS	sd	mg kg^-1^ TS	sd	mg kg^-1^ TS	sd	%		sd
I	Wheat straw	1	4,120	26.0	0.8	83.9	1.8	7.2	0.2	1.2	0.0	6,686.5	46.8	n.d.	.	74.3	15.4	2.2	20.0	0.1
I	Wheat straw	2	4,050	23.6	0.3	83.4	0.1	7.9	0.0	0.5	0.0	7,646.8	164.4	0.0	0.0	0.0	0.0	2.1	19.7	0.8
I	Corn stover	1	4,110	21.8	0.8	86.3	0.5	7.2	0.0	1.2	0.0	6,852.5	127.7	n.d.	.	0.0	0.0	2.2	19.9	0.3
I	Corn stover	2	4,110	27.8	2.4	82.4	1.1	6.9	0.0	0.5	0.0	10,743.8	5,758.5	0.0	0.0	0.0	0.0	1.9	21.8	1.5
F	Wheat straw	1	1,090	35.3	0.3	62.3	4.7	9.4	0.1	3.1	0.1	1,252.9	93.6	169.3	62.7	131.6	9.3	3.4	10.9	0.3
F	Wheat straw	2	910	46.2	0.5	65.3	1.5	9.1	0.0	1.8	0.0	873.2	52.4	113.0	31.7	9.5	0.3	3.4	10.2	0.0
F	Corn stover	1	1,000	48.8	2.2	73.1	0.6	9.4	0.1	4.3	0.1	742.9	21.2	109.5	16.7	198.4	1.5	3.2	12.8	0.1
F	Corn stover	2	970	50.4	0.5	69.8	0.7	9.0	0.0	1.8	0.1	992.7	29.7	108.3	24.6	5.4	0.8	3.3	11.2	0.0

I, Initial; F, Final; TS, total solids; VS, volatile solids; EC, electrical conductivity; TN, total nitrogen; C/N, carbon/nitrogen ratio; n.d., not determined.

### Greenhouse gas emissions and mass balance

**Fig 2:**
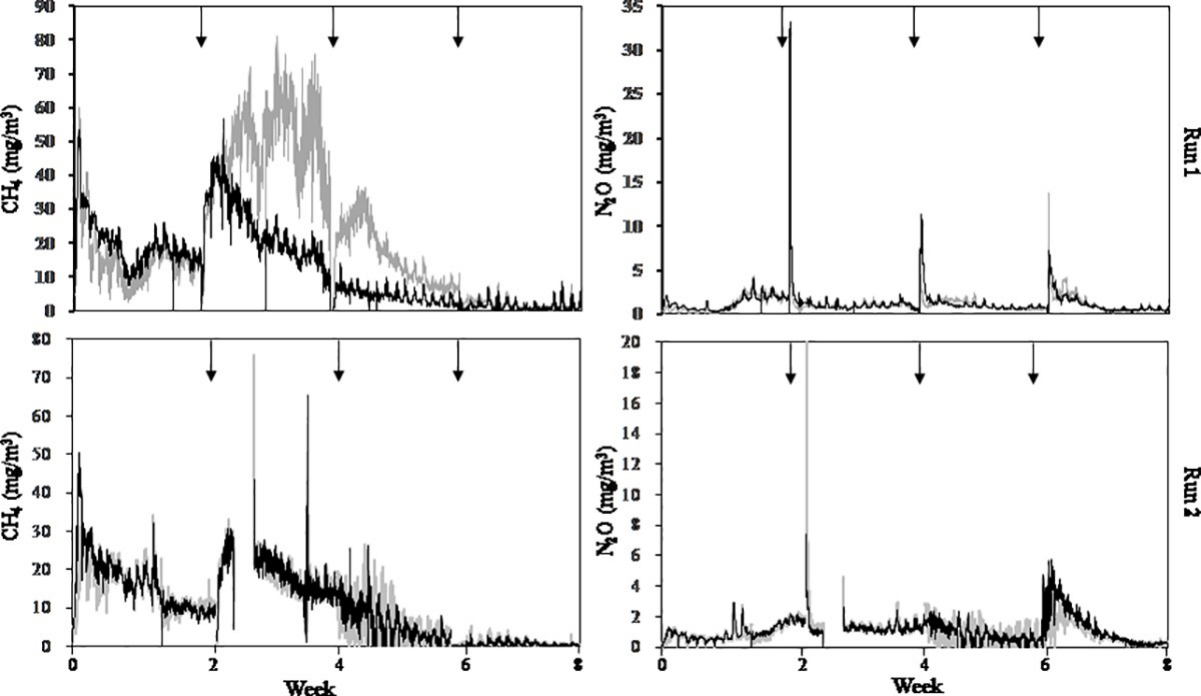
Methane (CH_4_) and nitrous oxide (N_2_O) emissions from compost piles with different bulking agents. Arrows indicate the pile turnings. Black lines indicate CS piles and gray lines indicate WS piles.

Gaseous emissions are described in Fig 2, and the mass balances of the composting experiments are summarized in Table 3. In these experiments, 35.4–45.0% of organic matter contained in the treated manure remained in the final product as mature compost. For nitrogen balance, 48.7–54.9% of initial nitrogen remained in the mature compost. The remaining 55.0–64.6% of initial organic matter or 35.4–45.1% of initial N contained in the treated manure was converted into gases such as CO_2_, CH_4_, NH_3_, N_2_O, and N_2_ and lost to the environment.

**Table 3:** Total mass balance in the composting experiments.

	CS		WS	
	kg	%	kg	%
Initial VS	733.6	100.0	726.0	100.0
Final VS	330.1	45.0	257.1	35.4
CH_4_	2.65	0.36	3.56	0.49
CO_2_	519.9	70.9	417.6	57.5
Initial N	29.8	100.0	29.0	100.0
Final N	16.4	54.9	14.1	48.7
N_2_O-N	0.17	0.58	0.16	0.54
NH_3_-N	2.74	9.18	1.52	5.25
Unknown	10.5	35.3	13.2	45.5

VS, volatile solids; Unknown, N loss that cannot be explained by N_2_O-N and NH_3_-N losses.

Methane emissions were highly disparate: 2,653.5±485.7 g (CS) and 3,558.1±1,996.4 g (WS). These values were equivalent to 0.36% (CS) and 0.49% (WS) of initial VS, respectively. Nitrous oxide emissions from the CS and TS piles were 173.5±20.8 g-N and 157.4±27.9 g-N, respectively, and these values were 0.58% (CS) and 0.54% (WS) of initial N. GHG emissions between the two bulking agents were not significantly different.

## Discussion

In this study, ear corn stover was collected for potential use as a bulking agent in dairy manure composting. The efficiency of collecting stover using a roll baler with or without a gyro rake was evaluated. The amount of corn stover left in the field was 7,612 kg ha^-1^, and its average moisture content dropped from 76.0% to 59.2% during the 2-week drying period. The collection experiment results showed that the use of a gyro rake increased the collection efficiency successfully from 22 to 26%, with lower diesel consumption and lower operation time. These values were significantly lower than those in a previous report in the United States, in which 50% collection efficiency was observed for a wet baling system [21]. Another work estimates 40% collection efficiency with a rake and 30% without [22], indicating there is room to improve the machinery to increase collection efficiency. Since it is known that 100% corn stover removal from the field can reduce grain yields and that removal of up to 50% is recommended for continuous production [23], future effort will be needed to improve stover collection efficiency.

The collected corn stover was air-dried and mixed with fresh dairy manure to make the compost. For comparison, wheat straw, which is frequently used as a bulking agent in local dairy systems in Hokkaido, was also used as a bulking agent for dairy manure composting. The CS piles showed a sufficiently high temperature during the 8-week process; the maximum temperature in the core zone reached 75.7±0.2°C, and 79.5±9.8% or 64.5±4.2% of the composting period showed a sufficiently high temperature (>60°C) in the pile core and bottom zone, respectively. This was comparable to the WS piles (maximum temperature 73.7±0.5°C), indicating that ear corn stover can be a satisfactory bulking agent for dairy manure composting. In a previous study with a small-scale reactor (effective volume 12.3 L) [23], the maximum temperature with ear corn residue was 54.3°C, and the temperature increase was slower than that of wheat straw, while another work with a similar pile scale (3.3 t) [24] showed that a similar temperature profile of over 70°C was achieved for a completely mixed pile, agreeing with our work especially in Run 2. It is well known that fresh animal manure contains human pathogens (*Escherichia coli* O157:H7, *Salmonella*, *Listeria*), plant pathogenic fungi (*Fusarium*, *Macrophomina*), or viruses (Tobacco mosaic virus or Tomato mosaic virus), and thermophilic composting can effectively reduce some of these pathogens in the final product [25,26]. Weed control is another major problem in organic farming, as is the use of manure compost [27], and our data with high pile temperature (>60°C) for 4 weeks is also enough to suppress seed viability effectively [28,29], even though parameters besides temperature can also affect seed viability [30]. Thus, a high pile temperature is required to kill both pathogens and weed seeds, which is important for the quality of final mature compost. A low enough moisture content of the final product (49.6±1.1% for CS piles and 40.7±7.7% for WS piles) also can contribute to re-germination of both pathogens and weed seeds.

Manure composting is also known to be a significant source of greenhouse gases such as methane (CH_4_) and nitrous oxide (N_2_O). Since the Japanese livestock production system operates on limited amounts of land, composting is a major process for treating livestock manure. Currently, 2,323 kt CO_2_ eq. (CH_4_) and 3,916 kt CO_2_ eq. (N_2_O) are estimated to be emitted by the Japanese livestock production system [31], and a mitigation strategy is needed to establish an environmentally friendly and sustainable production system. Previously we showed that the use of a bulking agent significantly reduces both CH_4_ and N_2_O emissions [18]. Therefore, corn stover can also be a good candidate for this strategy. In the present study, we used the same chamber system to estimate these GHG emissions, and the results show that CH_4_ emission (3.7±1.2 g kg^-1^ TS in CS piles and 4.8±2.3 g kg^-1^ TS in WS piles) and N_2_O emission (5.8±1.1 g N_2_O-N kg^-1^ N_initial_ in CS piles and 5.4±0.3 g N_2_O-N kg^-1^ N_initial_ in WS piles) were comparable for these treatments. A comparison with our previous study [18], using the same gas measurement methodology (chamber and continuous measurement with IPD), the same initial pile scale (4,000 kg), and the same experimental conditions (dairy cattle herd, feedstuffs, climate conditions, operating persons, and so on), shows that CH_4_ emissions from both CS and WS were significantly smaller than those with no additional control (20.8±1.3 g kg^-1^ TS) and even smaller than the piles with dried grass added (5.4±1.4 g kg^-1^ TS). This indicates that the use of a bulking agent (wheat straw, dried grass, or corn stover) can significantly reduce CH_4_ emissions through the active air (oxygen) supply into the pile, which potentially inactivates methanogen. Although the amount of bulking agent used in this study is smaller (150 kg) than in the previous one (400 kg, 10% w/w), enough of a mitigation effect on CH_4_ emission was achieved. For N_2_O emission, although the value obtained in this study was also lower than that of no additional control (7.4±2.6 g N_2_O-N kg^-1^ N_initial_), the mitigation effect was smaller than it was for the pile with dried grass. The potential explanation is that the temperature was significantly higher, above 70°C, which can suppress the nitrifier`s activity [32], but this seems not to be enough because the temperature of the pile with dried grass in our previous work was in the same range. Further research is needed to elucidate the N_2_O mitigation effect by the addition of different bulking agents.

Although the corn stover used in this study did not contain significant nitrate content (S1 File), it is known that fresh corn stem contains significant amounts of NO_3_-N, especially in the part close to the ground [33,34]. Nitrate accumulation in corn stem should be avoided because it is known to be toxic to dairy cattle [35], but it can also contribute to subsequent N_2_O production through denitrification. Mature compost, which is frequently used as an alternative bulking agent, also contains a large amount of NO_2_ or NO_3_-N and is known to contribute to N_2_O production at the initial stage of the composting process [36,37]. Therefore, the use of corn stover with high NO_3_^-^ content as a bulking agent should be avoided.

Another significant N loss during the composting process can occur through NH_3_ volatilization. The results show that CS piles have higher NH_3_-N emission than WS piles (Table 3). This might to be attributable to the higher maximum temperature in CS (75.7±0.2°C) than in WS (73.7±0.5°C), but the underlying mechanism is unclear. On the other hand, the final product (mature compost) of the CS pile was well N-conserved (16.4 kg; 54.9% of initial N) compared with WS (14.1 kg; 48.7% of initial N), indicating that total N loss during the process was lower when corn stover was used as the bulking agent (Table 3). This can present an advantage to using corn stover as a bulking agent since N will be used as the fertilizer for crop production thereafter.

In an integrated farming system of arable and livestock farms, efficiency is expected to be obtained by exchanging locally available resources (manure, wheat or rice straw, etc.). A shift from a linear to a circular production system that makes use of by-products is highly recommended from the point of view of environmentally friendly and sustainable agriculture [38,39]. Recently, large-scale farms with more efficient and economically favorable production systems have been expanding, while crop-integration systems that efficiently utilize N or C resources are declining worldwide [40]. A key issue is the efficient utilization of livestock manure in local production systems. The current economic circumstances surrounding Japanese commercial composting facilities that treat livestock manure are very severe. In many cases, production costs, such as the costs of electricity, bulking agent, or labor, can exceed the total sales of the final product (mature compost) [41]; a composting company in such a predicament cannot stand alone economically. The transport cost is also a bottleneck in manure spreading, and it is very rare for compost to be transported more than 30 km from the facility [41,42]. Therefore, manure should be processed and used within local production systems. The local utilization of ear corn stover has some potential to solve this problem, since sawdust or other biomass is now less available because of the use of these materials for energy production. Our data indicate that the use of corn stover can be at least an alternative bulking agent for manure composting without adverse effects on the environment, such as GHG emissions. However, economic studies to fix many problems, such as the cost of drying or transporting corn stover, remain to be conducted to assess the feasibility of using corn stover in local production systems.

In conclusion, ear corn stover can contribute to organic matter degradation and the mitigation of methane emission during the manure composting process. Corn stover can also make the pile temperature high enough to kill pathogens and weed seeds. These results clearly showed that the corn stover left after ear corn harvesting can be a good alternative bulking agent for dairy manure composting, which can lead to the development of more ecologically friendly dairy farming in the Hokkaido region. In future studies, the economic benefit or contribution of corn stover to local agricultural systems should be clarified to make it feasible for widespread use.

## Supporting information

S1 Fig Ammonia (NH_3_) and carbon dioxide (CO_2_) emissions from compost piles with different bulking agents. Arrows indicate the pile turnings. Black lines indicate CS piles and gray lines indicate WS piles.
(DOCX)

S1 File Chemical properties of the bulking agents. Raw data for Figs 1 and 2 and Tables 1–3 and S1.
(XLSX)
