# Supplementary material for: Use of corn stover as bulking agent in dairy manure composting toward Japanese circular dairy farming
Source: PLoS One. 2020 Oct 22;15(10):e0241064. doi: 10.1371/journal.pone.0241064 (PMC7580903; doi:10.1371/journal.pone.0241064)
Supplement: S1 Fig — Arrows indicate the pile turnings. Black lines indicate CS piles and gray lines indicate WS piles. (DOCX) [file pone.0241064.s001.docx]

**SUPPLEMENTAL INFORMATION**

**Use of corn stover as bulking agent in dairy manure composting toward Japanese circular dairy farming**

Koki Maeda*

NARO, Hokkaido Agricultural Research Center, Dairy Research Division, Sapporo, Japan

＊Corresponding author

Email: k_maeda@affrc.go.jp (KM)

Current address for KM:

JIRCAS, Crop, Livestock & Environment Division, Tsukuba, Ibaraki, Japan

Fig. S1. **Ammonia (NH_3_) and carbon dioxide (CO_2_) emissions from compost piles with different bulking agents.** Arrows indicate the pile turnings. Black lines indicate CS piles and gray lines indicate WS piles.
